# The pattern of *KRAS* mutations in metastatic colorectal cancer: a retrospective audit from Sri Lanka

**DOI:** 10.1186/s13104-017-2731-5

**Published:** 2017-08-10

**Authors:** Nirmala Dushyanthi Sirisena, Kemal Deen, Dayupathi Eranda Nipunika Mandawala, Pumindu Herath, Vajira Harshadeva Weerabaddana Dissanayake

**Affiliations:** 1 Human Genetics Unit, Faculty of Medicine, University of Colombo, Kynsey Road, Colombo 8, Sri Lanka; 2 Department of Surgery, Faculty of Medicine, University of Kelaniya, Kelaniya, Sri Lanka; 3 Asiri Centre for Genomic and Regenerative Medicine, Colombo 5, Sri Lanka

**Keywords:** Colorectal carcinoma, Epidermal growth factor receptor, Genotype, *KRAS* gene, Mutation

## Abstract

**Objective:**

Activating mutations in the *KRAS* gene, found in approximately 53% of metastatic colorectal cancer (mCRC) cases, can render epidermal growth factor receptor (EGFR) inhibitors ineffective. Regional differences in these mutations have been reported. This is the first study which aims to describe the pattern of *KRAS* mutations in a Sri Lankan cohort of mCRC patients.

**Results:**

The *KRAS* genotypes detected in mCRC patients which have been maintained in an anonymized database were retrospectively analyzed. Of the 108 colorectal tissue samples tested, 25 (23.0%) had *KRAS* mutations. Overall, there were 68 (63.0%) males and 40 (37.0%) females. Among the *KRAS* positive cases, there were 14 (56.0%) males and 11 (44.0%) females. Their age distribution ranged from 29 to 85 years with a median age of 61 years. There were 15 patients (60.0%) with point mutations in codon 12 while 10 (40.0%) had a single mutation in codon 13. The most common *KRAS* mutation identified was p.Gly13Asp (40.0%), followed by p.Gly12Val (24.0%). Other mutations included p.Gly12Cys (12.0%), p.Gly12Ser (12.0%), p.Gly12Asp (8.0%), and p.Gly12Arg (4.0%). The codon 13 mutation was a G>A transition (40.0%), while G>T transversions (32.0%), G>A transitions (24.0%), and G>C transversions (4.0%) were found in the codon 12 mutations. The frequency of *KRAS* mutations was similar to that reported for Asian patients. However, in contrast to several published studies, the G>A transition in codon 12 (c.35G>A; p.Gly12Asp), was not the most common mutation within codon 12 in our cohort. This may be a reflection of the genetic heterogeneity in the pattern of *KRAS* mutations in mCRC patients but valid conclusions cannot be drawn from these preliminary findings due to the small size of the study sample.

## Introduction

In Sri Lanka, colorectal cancer (CRC) is one of the top five cancers accounting for almost 7.0% of all male cancers and 6.0% of all female cancers [1]. Recent advances in molecular targeted therapeutic approaches to CRC have identified the potential role of anti-epidermal growth factor receptor (EGFR) targeted therapies, cetuximab and panitumumab as adjuvant treatment in advanced disease in combination with cytotoxic chemotherapy [2, 3]. However, EGFR, the target of these drugs, which is overexpressed in approximately 50.0–80.0% of CRC, failed to predict a therapeutic response when used clinically. Research showed that the Kirsten rat sarcoma viral oncogene homolog (*KRAS*) gene, an important member of the EGFR signalling cascade, can acquire activating mutations in exon 2 codons 12 and 13 in approximately 35.0–45.0% of the CRC cases, rendering EGFR inhibitors ineffective [4, 5]. In 2009, the US Food and Drug Administration (FDA) approved EGFR-targeted monoclonal antibody therapy with cetuximab and panitumumab in patients with metastatic colorectal cancer (mCRC) along with analysis of *KRAS* mutation status, which is a predictive biological marker of resistance [6–8].

Somatic *KRAS* mutations in codons 12, 13 (exon 2), 59, 61 (exon 3), 117 and 146 (exon 4) are common hotspots. Approximately 53.0% of patients with mCRC have *RAS* mutations, with 42.0% having *KRAS* exon 2 mutations and 11.0% having *KRAS* non-exon 2 and *NRAS* mutations [3]. Other non hotspot mutations in codons 2, 3, 4, 63 and 154 occur less frequently in CRC, accounting for less than 5.0% of all mutation types [3, 4]. Most of the reported mutations are single nucleotide point mutations, particularly G>A transitions and G>T transversions [9]. According to published western studies, among the codon 12 mutations, p.Gly12Asp, and p.Gly12Val are the most frequent, while in codon 13, the substitution of glycine for aspartate (p.Gly13Asp) is the most common [3]. Some new and uncommon *KRAS* mutations that are found in codons 45, 69 and 80 have also been identified in Chinese patients with CRC, but their clinical significance has yet to be defined [10, 11].

It is essential to investigate the underlying mutations in the *KRAS* gene in mCRC patients to identify the prevailing patterns as regional differences in these mutations have been reported in different population groups. Such knowledge would be helpful in selecting the appropriate patients with mCRC for EGFR-inhibitor therapy and for developing cost-effective targeted mutation testing for predominant mutations in the local context. This is the first study which aims to describe the frequency and types of *KRAS* mutations among Sri Lankan patients with mCRC referred for *KRAS* mutation testing to the only genetics diagnostic center undertaking such testing in the country.

## Main text

### Methods

The sex, age and *KRAS* genotypes detected in mCRC patients referred from all parts of Sri Lanka for *KRAS* mutation testing to the Asiri Centre for Genomic and Regenerative Medicine, Colombo from January 2010 to December 2014 have been maintained in an anonymized database completely de-identified from the original patients. This database was retrospectively analysed. All individuals listed in the database with mCRC who had undergone *KRAS* mutation testing during the period of study specified above were included in the retrospective analysis.

Genomic DNA was extracted from formalin-fixed paraffin-embedded (FFPE) tissue sections from histologically confirmed primary colorectal tumors using QIAamp DNA FFPE Tissue Kits [Qiagen, Germany (Cat No./ID: 56404)] according to the manufacturer’s protocol. The samples were specifically chosen by a pathologist to include predominantly tumor cells without significant necrosis or inflammation. The sequences of codons 12 and 13 of the *KRAS* gene were genotyped by polymerase chain reaction amplification and direct sequencing according to the validated method previously described by De Roock et al. [8]. This is a well-established test to detect mutations in codons 12 and 13 of the *KRAS* gene. In addition, in the diagnostic assay, internal positive and negative control samples were used and those samples gave the expected result always, indicating that the method used was 100% sensitive and specific. The primers and conditions for thermal cycling are available on request from the corresponding author. Cycle sequencing was performed with a BigDye Terminator Cycle Sequencing Ready Reaction kit (Applied Biosystems, Foster City, CA, USA). The sequences were read following capillary electrophoresis on an ABI 3130 Genetic Analyzer (Applied Biosystems, USA).

Data were analysed with respect to age groups, gender, and the *KRAS* genotype using standard descriptive statistics. Associations between variables were tested through Fisher’s Exact Test. All *p* values below 0.05 were considered statistically significant.

## Results

**Fig. 1:**
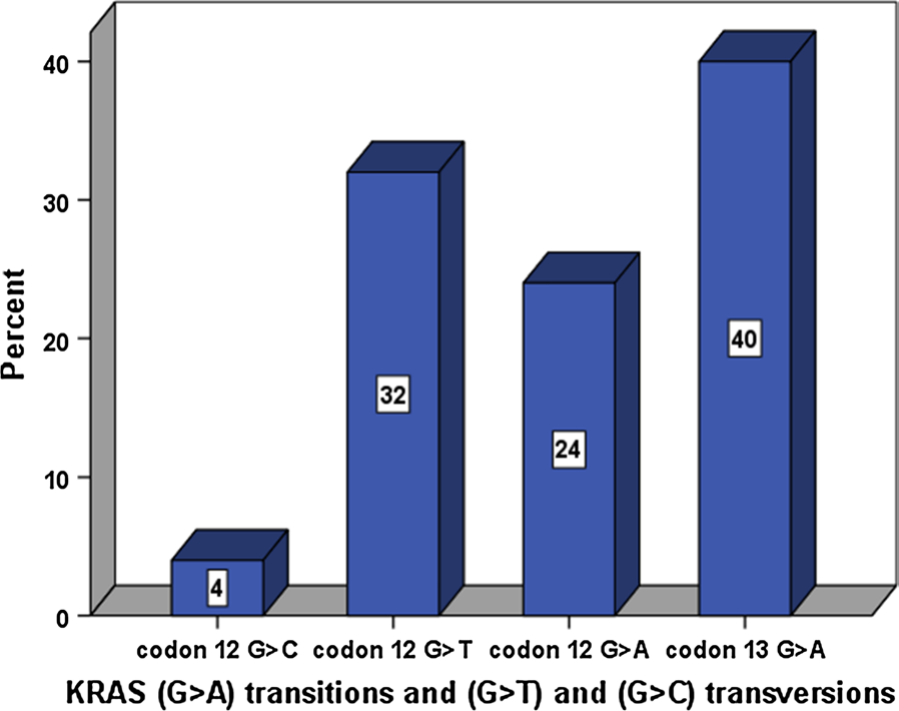
Percentage distribution of the mutant *KRAS* (G>A) transitions and (G>T) and (G>C) transversions in the study cohort

The colorectal tissue samples of 108 patients were tested. Of these, 25 (23.0%) tested positive for *KRAS* mutations. The remaining 83 (77.0%) samples had the wild-type allele. Overall, there were 68 (63.0%) males and 40 (37.0%) females. Among the *KRAS* positive cases, there were 14 (56.0%) males and 11 (44.0%) females. Their age distribution ranged from 29 to 85 years with a median age of 61 years. There were 15 patients (60.0%) with point mutations in codon 12 while 10 (40.0%) had a single mutation in codon 13. The most common *KRAS* mutation was NM_004985.3:c.38G>A; NP_004976.2: p.Gly13Asp (40.0%), followed by c.35G>T; p.Gly12Val (24.0%). The distribution of the different *KRAS* mutations identified in Sri Lankan mCRC patients is shown in Table 1. The G>A transitions in both codons 12 and 13 accounted for 64.0% of all the mutant *KRAS* cases. Figure 1 shows the percentage distribution of the mutant *KRAS* (G>A) transitions and (G>T) and (G>C) transversions identified in the Sri Lankan cohort.

**Table 1:** Distribution of *KRAS* mutations in Sri Lankan colorectal cancer patients

*KRAS* mutation type	Number of patients (*n*)	Percentage (%)
Codon 12		
p.Gly12Cys (c.34G>T)	3	12.0
p.Gly12Val (c.35G>T)	5	20.0
p.Gly12Val (c.35G>A)	1	4.0
p.Gly12Arg (c.34G>C)	1	4.0
p.Gly12Ser (c.34G>A)	2	8.0
p.Gly12Ser (c.35G>A)	1	4.0
p.Gly12Asp (c.35G>A)	2	8.0
Codon 13		
p.Gly13Asp (c.38G>A)	10	40.0
Total	25	100.0

**Fig. 2:**
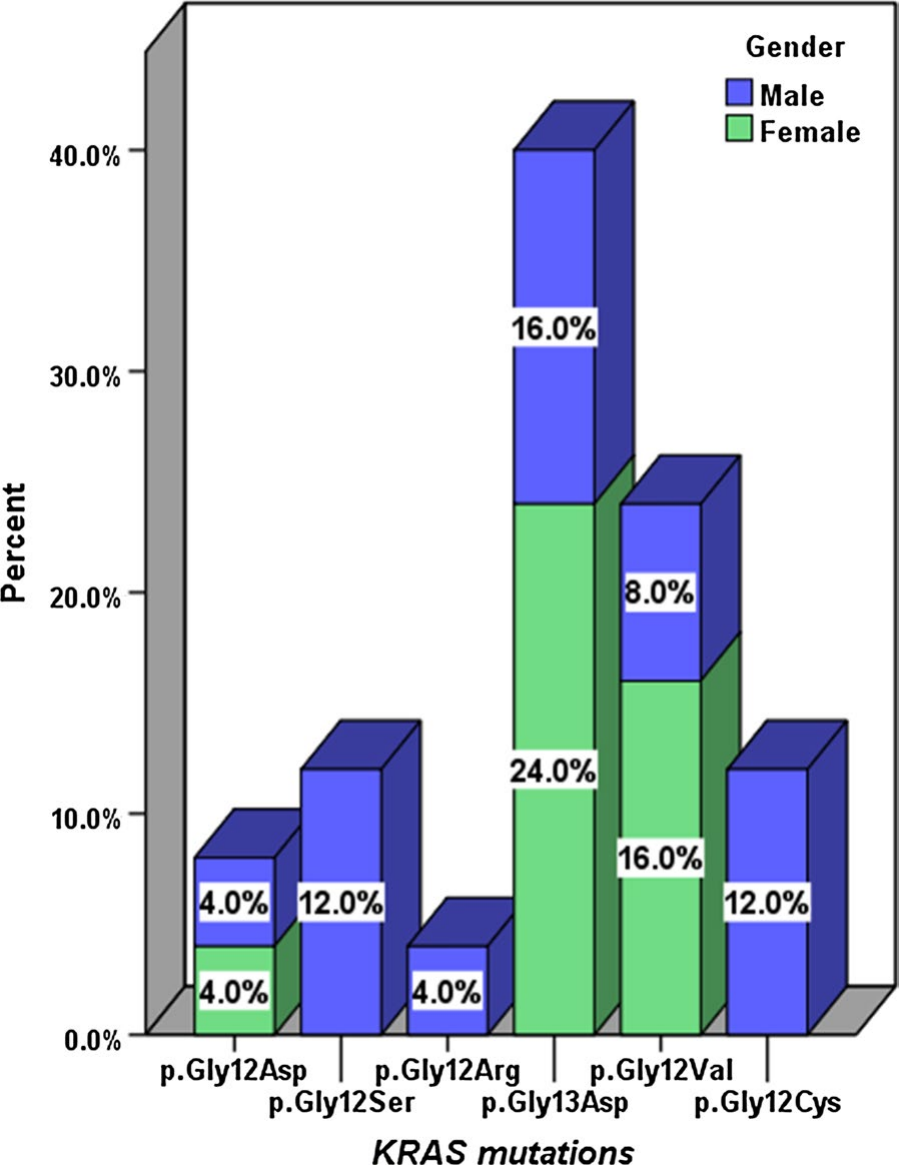
Percentage distribution of the *KRAS* mutations according to gender

**Fig. 3:**
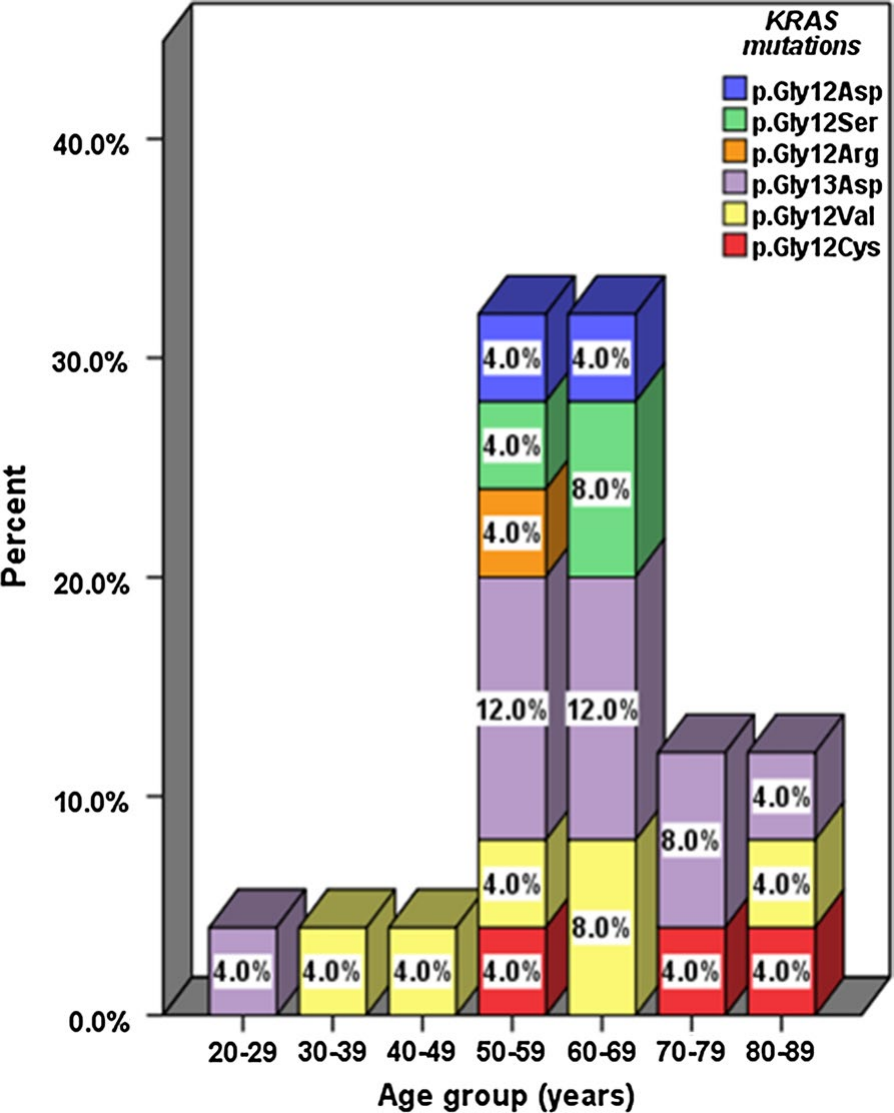
Percentage distribution of the mutant *KRAS* mutations according to age groups

The *KRAS* mutation pattern according to gender correlated well with the overall *KRAS* mutant type. The p.Gly13Asp (c.38G>A) mutation had the highest frequency in both males (16.0%) and females (24.0%). The gender distribution of the *KRAS* genotypes is shown in Fig. 2. No statistically significant difference was found between the *KRAS* genotypes and the gender (*p* = 0.153, Fisher’s exact test). The highest percentage of mutant *KRAS* cases was found in the 50–59 years age group. The stratification of the mutant *KRAS* genotypes according to age groups is shown in Fig. 3. No statistically significant difference was found between the *KRAS* genotypes and the age groups (*p* = 0.981, Fisher’s exact test). Multiple mutations in the same individual were not detected in any of the patients in this cohort.

## Discussion

The *KRAS* mutation frequencies in Asian, European, and Latin American mCRC patients were reported to be 24.0, 36.0 and 40.0%, respectively [2]. The overall frequency (23.0%) observed in this study is in accordance with what is reported for Asian patients. It is unclear why a relatively lower frequency is observed in Asian patients. The difference of mutation status may result from various factors, such as the FFPE tissue samples, the percent of tumor cells, the extracted DNA quality, the testing methods and the testing target as well as different molecular pathogenetic mechanisms and environmental exposures [12].

Previous studies [2, 6, 7] have reported that the most common *KRAS* mutation types are p.Gly12Asp, p.Gly12Val and p.Gly13Asp, accounting for almost 70.0% of all mutations. Overall, a similar pattern was seen in this study where these 3 mutations accounted for 72.0% of all the *KRAS* mutations. The most common single mutation (40.0%) identified in our cohort was a G>A transition in codon 13 (c.38G>A; p.Gly13Asp), as opposed to mutations in codon 12 which have been reported in several Caucasian and Asian studies. Furthermore, our findings indicated a relatively lower frequency (8.0%) of the G>A transition in codon 12 (c.35G>A; p.Gly12Asp). However, it should be noted that our sample size was too small to draw meaningful conclusions regarding these variations. A larger study with sufficient numbers would be required to derive meaningful results to determine whether these variations are valid.

The frequency of *KRAS* mutations among 99 Japanese mCRC patients was reported to be 37.4% (37/99) [2]. Similar to our findings, the most prevalent mutation within codon 13 was the GGC→GAC (p.Gly13Asp) mutation found in 11 (29.7%) patients. However within codon 12, they obtained a higher frequency of the GGT→GAT (p.Gly12Asp) mutation found in 10 (27.0%) patients, followed by the GGT→GTT (p.Gly12Val) found in 8 (21.6%) patients. A similar pattern was reported in a Chinese study where *KRAS* mutations were detected in 33.3% (30/90) of the CRC tumor samples using the DNA sequencing method [3]. In a study conducted among Tunisian patients, *KRAS* somatic mutations were detected in the CRC tissue samples of 31.5% (16/51) patients [13]. According to their findings, 81.2% had a single mutation in codon 12 and 23.0% had a single mutation in codon 13. In contrast to our findings where transitions accounted for 64.0%, 81.3% of their mutations were transitions and 23.0% were transversions and the most common single mutation (50.0%) was a G>A transition in codon 12 (c.35G>A; p.Gly12Asp). A preponderance of the G>A transition in codon 12 (c.35G>A; p.Gly12Asp) has been reported in several other Caucasian and Asian studies [12, 14–19]. In contrast to these studies, the most common mutation identified in codon 12 in this study was p.Gly12Val (24.0%). A similar finding was observed in a retrospective study conducted among 299 patients with mCRC in India [7]. This may be a reflection of the genetic heterogeneity in the pattern of *KRAS* mutations in mCRC patients. However as stated earlier, valid conclusions cannot be drawn regarding these variations due to the limitations of this study.

Another factor that may influence the results is the method of detection and the sample type. Pyrosequencing, a real-time, non-electrophoretic, nucleotide-extension sequencing and next generation sequencing techniques, have been shown to be efficient in various applications in comparison to direct sequencing. A study demonstrated that pyrosequencing detected 17.9% of the *KRAS* mutations in patients with *KRAS* wild-type using direct sequencing alone (30 tumors among 168) [20]. It is believed that the presence of subclones harboring *KRAS* mutations within genetically heterogeneous tumors may explain the low-frequency *KRAS* mutations detected by the direct sequencing method [21]. It is important to note that approximately 20.0% of patients who test wild-type based on *KRAS* exon 2 analysis may actually harbour undetected extended *RAS* mutations in codons 59, 61 (exon 3), or codons 117 and 146 (exon 4) [22, 23].

Some studies have reported significant differences between the *KRAS* genotypes and clinicopathological variables such as age, gender, tumour location, tumour histopathology, and metastasis while other studies observed no significant effects [7, 11]. Similar to the findings in the Indian study reported by Veldore et al. [7], no significant correlation was found between the *KRAS* genotypes and the patients’ age and gender in this study. However, significantly higher mutation rates in well-differentiated tumours and tumours located at the distal end of the colon were observed in the Indian cohort.

## Limitations

Our preliminary findings suggest a frequency of the *KRAS* mutation at 23.0% indicating that this testing is crucial for targeted therapy management in mCRC in Sri Lanka. Using *KRAS* testing to restrict the use of EGFR-inhibitor therapy would help to select the appropriate patients and avoid administration of unnecessary, ineffective and toxic treatments to patients who would not benefit from them. However, the present study had a number of limitations. Most importantly, it was a retrospective study, the sample size was small and clinico-pathological parameters of the tumour were not available for analysis. In addition, other biomarkers, such as *KRAS* exon 3 or 4, *NRAS*, or *BRAF* mutations were not evaluated. It is recommended that future studies be undertaken with larger samples to correlate the *KRAS* genotype with the clinicopathological parameters in Sri Lankan patients with mCRC. Analysis of other homologues such as *NRAS* and downstream signalling effectors such as *BRAF*, would also be valuable.

## Abbreviations

mCRC: metastatic colorectal cancer (mCRC)
EGFR: epidermal growth factor receptor
FFPE: formalin-fixed paraffin-embedded
